# Proactively tailoring implementation: the case of shared decision-making for lung cancer screening across the VA New England Healthcare Network

**DOI:** 10.1186/s12913-023-10245-9

**Published:** 2023-11-22

**Authors:** Abigail N. Herbst, Megan B. McCullough, Renda Soylemez Wiener, Anna M. Barker, Elizabeth M. Maguire, Gemmae M. Fix

**Affiliations:** 1 Center for Healthcare Organization & Implementation Research, VA Bedford Healthcare System, 200 Springs Road (152), Bedford, MA 01730 USA; 2 Department of Public Health, Zuckerberg School of Health Sciences, University of Massachusetts, Lowell, MA USA; 3 National Center for Lung Cancer Screening, Veterans Health Administration, Washington, DC, US USA; 4 The Pulmonary Center, Boston University Chobanian &, Avedisian School of Medicine, Boston, MA USA; 5 General Internal Medicine, Boston University Chobanian & Avedisian School of Medicine, Boston, MA USA

**Keywords:** Lung Cancer Screening, Shared Decision-Making, Patient-centered Care, Implementation Science, Implementation Modifications, Ethnographic Research Methods

## Abstract

**Background:**

Shared Decision-Making to discuss how the benefits and harms of lung cancer screening align with patient values is required by the US Centers for Medicare and Medicaid and recommended by multiple organizations. Barriers at organizational, clinician, clinical encounter, and patient levels prevent SDM from meeting quality standards in routine practice. We developed an implementation plan, using the socio-ecological model, for Shared Decision-Making for lung cancer screening for the Department of Veterans Affairs (VA) New England Healthcare System. Because understanding the local context is critical to implementation success, we sought to proactively tailor our original implementation plan, to address barriers to achieving guideline-concordant lung cancer screening.

**Methods:**

We conducted a formative evaluation using an ethnographic approach to proactively identify barriers to Shared Decision-Making and tailor our implementation plan. Data consisted of qualitative interviews with leadership and clinicians from seven VA New England medical centers, regional meeting notes, and Shared Decision-Making scripts and documents used by providers. Tailoring was guided by the Framework for Reporting Adaptations and Modifications to Evidence-based Implementation Strategies (FRAME-IS).

**Results:**

We tailored the original implementation plan to address barriers we identified at the organizational, clinician, clinical encounter, and patient levels. Overall, we removed two implementation strategies, added five strategies, and modified the content of two strategies. For example, at the clinician level, we learned that past personal and clinical experiences predisposed clinicians to focus on the benefits of lung cancer screening. To address this barrier, we modified the content of our original implementation strategy *Make Training Dynamic* to prompt providers to self-reflect about their screening beliefs and values, encouraging them to discuss both the benefits and potential harms of lung cancer screening.

**Conclusions:**

Formative evaluations can be used to proactively tailor implementation strategies to fit local contexts. We tailored our implementation plan to address unique barriers we identified, with the goal of improving implementation success. The FRAME-IS aided our team in thoughtfully addressing and modifying our original implementation plan. Others seeking to maximize the effectiveness of complex interventions may consider using a similar approach.

## Contributions to the literature

• Shared Decision-Making for Lung Cancer Screening presents a unique implementation challenge; Lung Cancer Screening is the only preventive service for which the Centers for Medicare and Medicaid requires a conversation about benefits and harms for reimbursement.

• Traditional approaches to developing implementation plans may fall short in implementing Shared Decision-Making for Lung Cancer Screening, given the complexity of eliciting and responding to patient values during time-limited appointments.

• Tailored implementation plans improve the fit of implementation efforts to specific contexts. We applied the Framework for Reporting Adaptations and Modifications to Evidence-based Implementation Strategies (FRAME-IS) to proactively guide tailoring of our implementation plan.

## Background

Lung cancer is the leading cause of cancer death among both the general population and Veterans [1, 2]. In randomized trials, lung cancer screening (LCS) with low-dose chest computed tomography (CT) has been shown to reduce lung cancer mortality by 20% [3, 4]. LCS carries the potential benefit of saving lives due to early detection and treatment of lung cancer, but there are also potential harms. Harms include distress related to screen-detected findings, radiation exposure, invasive procedures that may cause physical complications, and overdiagnosis [5, 6]. Consequently, the United States Preventive Services Task Force (USPSTF), the American Thoracic Society (ATS), and the Department of Veterans Affairs (VA), among others, recommend Shared Decision-Making (SDM) for LCS [5–12]. Further, the Centers for Medicare and Medicaid Services (CMS) requires a documented SDM encounter for LCS reimbursement [13, 14]. SDM for LCS is notable; it is the first time CMS has instituted an SDM requirement for a preventive healthcare service [14, 15]. SDM for LCS may confer an additional benefit in supporting adherence to annual screening, as has been shown in other cancer screening contexts [16] though further study is warranted in this area [17].

SDM is a process in which patients and clinicians work together to make medical decisions that align with the patient’s preferences, values, and goals, as well as the clinical evidence [18]. SDM involves *information sharing*, *deliberation*, and the resultant *decision* [19]. USPSTF recommends the conversation include information about the benefits, limitations, and harms of LCS [20].

**Fig. 1:**
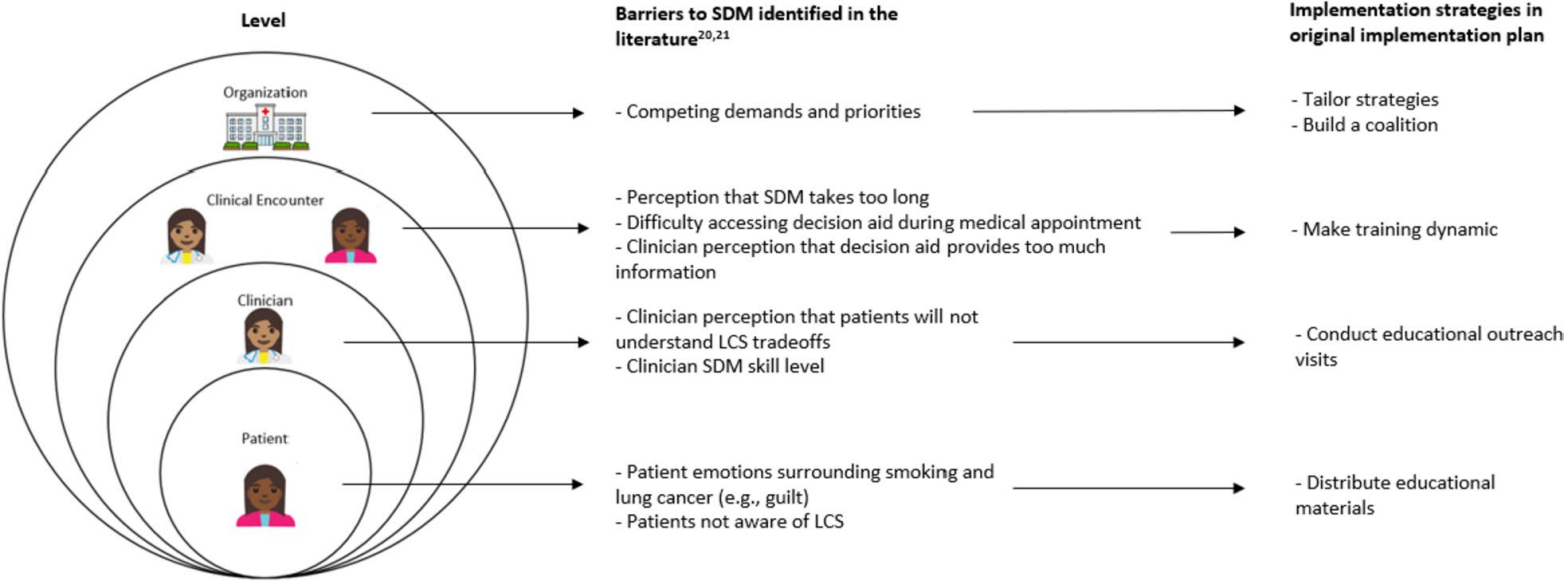
Socio-ecological model illustrating established barriers to SDM used in the creation of the original implementation plan and the implementation strategies selected for the original implementation plan

Guideline-concordant SDM for LCS does not routinely occur in practice [21–23]. Barriers to SDM for LCS exist at multiple levels of influence. Thus, we selected the socio-ecological model (Fig. 1) to stratify barriers [24]. Innovative approaches tailored to the local context are needed to achieve implementation of SDM for LCS in practice [17]. Our project, Whole Health Approach to Implementing Shared Decision Making for Lung Cancer Screening (WISDOM LCS), aims to increase guideline-concordant LCS, namely, the frequency and quality of SDM for LCS, in the VA New England Healthcare System over the five-year grant period. Notably, VA is transforming to a patient-centered, Whole Health (WH) approach to care, that includes centering clinical care around Veterans’ values and life goals [25]. With WISDOM LCS, we seek to leverage VA’s transformation to the WH approach to implement SDM. WISDOM LCS is organized around the socio-ecological model and includes intervention components at the organization, clinician, clinical encounter, and patient levels to address these multi-level barriers. To create our original implementation plan in the grant-writing stage, we conducted a brief literature review to identify activities that were effective in other settings to overcome known barriers to SDM identified in the literature. We used this information and drew from the “Expert Recommendations for Implementing Change” (ERIC) compilation of implementation strategies to formulate our original implementation plan [26].

As tailoring is important for implementation strategies to be effective, we sought to proactively tailor our implementation plan [27–29]. As an early step in WISDOM LCS, prior to implementation, we conducted a formative evaluation to understand barriers to SDM for LCS across VA New England. We used the Framework for Reporting Adaptations and Modifications to Evidence-based Implementation Strategies (FRAME-IS), a tool to document implementation plan modifications [30]. Implementation science recognizes the importance of local context to the uptake of evidence-based practices. Much work has been devoted to how to design and document modifications [31–33], but more attention to the nature and extent of modifications is needed [30, 34]. We sought to understand the local context to proactively tailor our original implementation plan to achieve guideline-concordant LCS. This manuscript offers an emic, “inside look” into the process of collecting data to make planned modifications to an implementation plan.

## Methods

We used an ethnographic study design to conduct a formative evaluation to identify barriers to SDM and inform the tailoring of our implementation plan. Data consisted of semi-structured qualitative interviews, fieldnotes of team meetings, and document review. The study setting was the VA New England Healthcare System. The local Institutional Review Board (IRB) designated this work as IRB-exempt, thus, this study was approved and overseen by the VA Bedford Research and Development Committee. All methods were carried out in accordance with relevant guidelines and regulations. Informed verbal consent was obtained from all subjects.

### Data collection

We interviewed leadership tasked with implementing LCS and clinicians involved in LCS. We used a purposeful sampling strategy based on role. Beginning with a list of LCS coordinators at each facility, we used snowball sampling to identify other staff involved in LCS, such as primary care providers. Seven of the eight VA New England medical centers had an LCS coordinator, with variation in specific responsibilities at the different facilities. Responsibilities could include enrolling patients in LCS, conducting SDM, and/or coordinating evaluation of screen-detected findings. Potential participants were contacted via email to invite interview participation.

Interviews occurred by phone, and covered the local facility LCS program, perceptions of SDM for LCS, and clinician perceptions of their role in the LCS process. The interview guide was designed to be used flexibly, depending on the participant’s background, and follow the course of the conversation. If, during the interviews, participants mentioned documents they used to support LCS, we asked for a copy. We audio-recorded interviews with each participant’s assent, between February and November 2020. Additionally, we attended one VA New England-wide LCS coordinator meeting where SDM was discussed. One team member took detailed fieldnotes of the meeting content. The meeting facilitator’s minutes were also obtained.

### Data analysis

Data was analyzed using an ethnographic approach which brings different data sources together to “layer” them for a “thick description” of the context, including barriers [35, 36]. The data (interviews, documents, fieldnotes, and minutes) were analyzed using a two-step process. Summaries of the interview audio were organized in Excel; categories included “provider perception of role” and “intent of LCS program” [37]. One team member who did not conduct the interview listened to the audio and recorded key responses, including verbatim text. The summaries were then reviewed by the interviewer. Deductive coding, based on study goals and commonly known barriers to SDM for LCS, such as appointment time constraints, were used to identify perceptions of and barriers to SDM in practice. Inductive coding was used to identify emergent themes. Using an iterative process, we further summarized data across participants. Then the full team used the socio-ecological model to organize barriers at the organization, clinician, clinical encounter, and patient levels [24]. Consensus was reached for each step.

We then organized these findings to inform our implementation plan modifications using the FRAME-IS [30].The FRAME-IS provides implementation scientists with modules to review and modify implementation plans. Based on the framework, we tailored the implementation plan to address the identified barriers at each level. As such, we considered the content of the modification and the nature of the modification at each level. Research team members consisted of anthropologists (GMF, MBM), health communication researchers (ANH, AMB, EMM), and a pulmonologist (RSW). The pulmonologist on our team is also co-chair of the VA New England LCS Council.

## Results

We interviewed 15 key people across seven VA New England medical centers, including regional and facility leadership, primary care providers, pulmonologists, a tobacco cessation psychologist, and LCS coordinators. Additionally, we observed one regional team meeting and collected six documents about LCS. These documents included one LCS script, one risk summary document, meeting minutes from one meeting, clinical reminder text from two sites, and an LCS decision aid. The observation and fieldnote data were used to describe the context of each barrier below. An eighth facility did not have an LCS coordinator or LCS program in place; no other participants were identified at this facility.

### VA New England context

We learned about the LCS program history and structure in the region, key contextual information important to implementation. LCS programs were supported by the VA New England Healthcare System, including two-year start-up salary support funds for LCS coordinators, with the expectation that facilities would cover future salary support. LCS coordinators were nurses, nurse practitioners, or physicians’ assistants and were not required to have background in LCS prior to the role. VA New England was further along in implementing LCS than many other regions, measured by percentage of patients screened [38].

From the interviews we learned that of the eight VA New England medical centers, five facilities had a decentralized LCS program model, two facilities had a centralized LCS program model, and one facility did not have an LCS program [39]. In decentralized models, conversations about LCS, and ordering LCS exams, occurred in primary care. In centralized models, patients were referred by primary care to an LCS program. In this latter model, conversations about LCS took place over the phone with an LCS coordinator and/or included a medical record review to confirm LCS eligibility. There was variation within and across these LCS program models; three facilities had an LCS coordinator but did not have a “clinical reminder” for LCS—a prompt in the electronic health record that identifies the patient as eligible for certain healthcare services and reminds the clinician to offer the service during the appointment [40].

### Barriers and adaptations

Across both the centralized and decentralized programs, we identified barriers to SDM at the organization, clinician, clinical encounter, and patient levels. Based on these findings, we adapted our original implementation approach. Below we summarize the barriers at each level followed by a description of the tailored implementation strategies to address the barriers.

In Table 1, we summarize our original implementation plan, the barriers identified at each level, and how each level was tailored to address these barriers.

**Table 1:** Original implementation plan, barrier, and tailored implementation plan by barrier of the socio-ecological model

Level	Original Implementation Strategy	Key Themes from Interviews	Tailored Implementation Strategy
Organization	Tailor strategies • Conduct bi-annual conference calls with stakeholder council to develop stakeholder-guided implementation plan Build a coalition • Align with ongoing WH initiative to strengthen implementation effort	LCS is not prioritized at facilities • Leadership is focused on other services and healthcare needs, such as colonoscopy • Regional leadership has not communicated sufficiently to clinicians about SDM or LCS • Clinical reminder for LCS is not activated at all facilities • Limited knowledge of WH initiative	Tailor strategies Revise professional roles • LCS Coordinator responsible for SDM • Recommend to regional LCS Council that LCS Coordinator should be responsible for SDM Identify and prepare champions • Advocate for replicating model of exemplar facility, where LCS coordinator is responsible for SDM in meetings with regional medical leadership • Meet with peer leader of LCS coordinators to brainstorm strategies to disseminate new role responsibilities to colleagues across region Use advisory boards and workgroups • Identify overlap between WH and SDM • Brainstorm WH-inspired messaging • Infuse WH initiative messages into SDM implementation
Clinician	Make training dynamic • Training event for LCS coordinators and interested PCPs	Personal and clinical experiences inform clinician recommendations for screening • Pro-screening bias • Perception that SDM is the same as education	Make training dynamic Develop training curriculum to explicitly address clinicians’ personal experiences • Include self-reflection about beliefs about screening • Clarify that SDM is dynamic and incorporates patients’ values
Clinical Encounter	Conduct educational outreach visits • Academic detailing to disseminate LCS personalized decision support tool	Perception that LCS is straightforward and akin to recommending other screenings. Thus, information, like that in decision aids, is unnecessary • Belief that SDM is happening already • Lack of information sharing about harms of LCS • Decision aids are inaccessible and/or redundant	Conduct educational outreach visits • Address provider-specific barriers during academic detailing; demonstrate how SDM is nuanced and based on the individual patient • Include opportunities to practice SDM during one-on-one sessions • Present screenLC.com, a web-based decision support tool for point-of-care use, to LCS coordinators
Patient	Distribute educational materials • Adapt existing patient-facing educational materials about LCS	Clinicians perceive that patient role in decision is adequate • Perception that patients are content with current LCS process • Perception that patients’ past decision to smoke precludes them from making a decision about LCS	Involve patients and family members • Develop plan to incorporate patients actively in SDM process Develop educational materials • Co-design patient-facing materials with Veterans and clinicians that empower Veterans to ask questions during medical appointments • Address smoking stigma explicitly in patient-facing materials

^*^Implementation strategies are underlined for clarity

### Original implementation plan: organizational level

We initially developed an implementation plan with the ERIC implementation strategies *Tailor Strategies* and *Build a Coalition* at the organizational level. Our original plan included convening a council to develop a stakeholder-guided implementation plan, tailoring strategies based on our formative evaluation, as described in this manuscript. We also planned to align our implementation efforts with VA’s patient-centered, WH initiative, by building a coalition of WH champions and stakeholders.

### Organizational-level barriers

Participants described several organizational barriers to SDM, including: 1) Being unaware of Whole Health; 2) Lack of communication about LCS goals or structure; 3) Perceived pressure to demonstrate the value of the LCS coordinator role; 4) Insufficient priority of LCS in relation to competing demands; 5) Time constraints in primary care; 6) No systematic prompts to trigger LCS discussions.

#### Unaware of Whole Health

Participants had little or no knowledge of VA’s WH initiative: *“I’ve heard of Whole Health; I don’t really know what Whole Health is.”* – Tobacco Cessation Counselor (TCC) 01. Clinicians believed that SDM and WH were aligned but were unfamiliar with WH prior to the interview.

#### Lack of communication about LCS goals or structure

Participants noted that the VA New England leadership had not conveyed the goal of the LCS program: *"We don't receive any messaging” –* LCS Leadership (LCSLead) 01. Another lead described how the overall LCS program was comprehensive but did not give specific direction about what tasks to prioritize. Leadership, including participants interviewed, wanted to balance standardizing the program with allowing facilities to adapt to their needs: *“Have some flexibility to decide, ‘how are you going to use your coordinator?’ Maybe we have actually erred too much on the side of letting facilities do their own thing” –* LCSLead02. Yet this flexibility led to confusion about how to implement elements of the LCS program, including SDM.

#### Pressure to demonstrate value

Several LCS coordinators commented that they hoped funding for the program would continue and that they felt pressure to enroll as many patients as possible into the screening program to demonstrate the value of their position: *“From my understanding the funding for my position was provided from the* [regional] *level, I believe, for a certain period of time and then after that we are hoping to provide the workload credit and justification to keep me permanently.”* – LCS Coordinator (LCSC) 03. They perceived LCS uptake as a more tangible metric than SDM for LCS.

#### Insufficient priority of LCS

Facility leadership did not consistently prioritize LCS relative to other initiatives. This was attributed in part to the patient population being unaware of LCS; therefore, there was little demand from patients for screening. One LCS lead went on to explain: *“We have so many things that are a priority right now and so many pressures on us….That’s one thing that probably went into the calculation, the lack of [patient] demand.” –* LCSLead02. Others raised concerns about the value of LCS relative to other healthcare services. *“I have not detected a suspicious nodule that has gone on to be malignant. My impression is that we do an awful lot of CT scans to detect one malignancy…We have limited funds to hire staff, so we have to make decisions about what programs are the most important. I’m not saying this program’s not important, but it has to be weighed in the costs versus benefits.” –* Primary Care Leadership (PCLead) 02.

#### Primary care time constraints

Brief primary care visits did not allow clinicians time for SDM. A coordinator at a facility with a decentralized LCS model noted primary care: *“[has] got a 20–30 min appointment, in which you have to review all their meds, make sure their meds are up to date…all their immunizations, all their health things like colonoscopies, mammograms, and then you have to have the conversation, yes, you are still smoking, and then they have to find out the pack-years and do all that stuff in order to sign them up [for LCS]. A lot of people don’t have time to do that.”* – LCSC06.

#### No systematic prompts

Despite all facilities being directed by VA New England leadership to implement the LCS clinical reminder, not all facilities did. A pulmonologist noted his training served as a reminder in the absence of reminders at his facility: *“when I see a patient who meets eligibility, [LCS] comes to mind immediately, as ‘did we check this box?’ I’m not getting reminders.”* – Pulmonologist (PULM) 01.

### Organizational-level tailoring

To address facilities’ inconsistent prioritization of LCS in relation to other clinical issues, we modified our implementation plan to include an additional ERIC strategy: *Revising Professional Roles* of LCS coordinators to shift SDM responsibility to them. We initially intended to target primary care providers (PCP), but we subsequently adapted our approach to target a new, more focused, population: LCS coordinators. To complement the strategy *Revise Professional Roles*, we also added the ERIC strategy *Identify and Prepare Champions* to facilitate the revision of LCS coordinator roles. We engaged regional leadership, including regional LCS leadership, to secure their willingness to actively support and participate in revising the LCS coordinator role. We also modified our plan by replacing the strategy *Build a Coalition* with the strategy *Use Advisory Boards and Workgroups.* Specifically, we engaged WH experts in both our stakeholder council and smaller working groups to help us conceptually and practically integrate WH and SDM and address the lack of WH awareness among some VA New England clinicians.

### Original implementation plan: clinician level

Our original implementation plan consisted of the implementation strategy *Make Training Dynamic* at the clinician level. We planned to hold a training event for LCS coordinators and PCPs.

### Clinician-level barriers

Participants described several clinician-level barriers to SDM for LCS: 1) Personal experiences that influence their thinking about LCS; 2) Conflation of SDM and patient education; 3) Perceived value of LCS limiting need for SDM; 4) Comparison to other preventive screenings, which were not thought to have an SDM recommendation.

#### Personal experiences influencing LCS conversations

LCS coordinators, PCPs, and pulmonologists described past experiences as influencing the way they approached LCS conversations with patients. Experiences related to lung cancer weighed heavily on clinicians and impacted the way they approached LCS conversations: *“I have a bias, I have a personal family history of lung cancer, so I would probably err on the side of screening”* – LCSC01.

Concern about a missed cancer diagnosis prompted many clinicians to encourage LCS. One participant described an experience where a patient had a delayed follow-up screening: *“We did have a bad outcome…there was a recommendation that came in for a follow-up CT, a CT was ordered, but due to COVID, they weren’t doing CT scans, so the follow-up CT scan was probably 6-plus months late and by then the patient had metastatic cancer”* – PCLead02.

Comparatively, another LCS coordinator talked about the satisfaction in identifying cancers*. “Out of [number of Veterans screened] …we had [several] cancers that were treated. That makes me happy.”* – LCSC06.

#### Conflation of SDM and patient education

SDM should entail a conversation of the benefits, harms, and eliciting patient values about screening. When we asked about SDM, clinicians often spoke about education instead. *"Providers feel that there is a push towards this [SDM] and the importance of it, and we all know education is important"* – LCSC03.

#### Perceived value of LCS limiting need for SDM

Many participants characterized SDM conversations as convincing patients to get screened: *“I wish that the shared decision-making process was a little more geared towards how can we make patients understand that this is a valuable thing…I wish there was a little more encouragement in the process, rather than ‘are they happy with the decision that they made’”* – LCSC02 This participant went on to describe the benefit of screening and thus, the purpose of her job: *"I want to enroll as many patients as I can because I want to find the cancer.”* – LCSC02.

Similarly, another implied that getting screened was the clinically correct choice. *"The data supports so strongly that [LCS] is beneficial, that it doesn't seem like there's much of a decision.”* – LCSC08.

#### Comparison to other preventive screenings

Others were unaware of SDM being recommended for other screenings; therefore, participants questioned its value for LCS. During an LCS team meeting, we captured a discussion where the LCS coordinators were unsure how LCS compares to other cancer screening tests and wondered why SDM isn’t recommended for colonoscopy.

### Clinician-level tailoring

We learned providers’ personal and clinical experiences impacted their perception of SDM. We thus tailored our training curriculum of the ERIC strategy *Make Training Dynamic*. To address clinician-level barriers, our training curriculum for LCS coordinators explicitly addressed personal experiences related to LCS. We tailored our training to include self-reflection modules that focused on beliefs about screening and lung cancer. To address the conflation of SDM with patient education and the belief that the value of LCS limits the need for SDM, we defined the components and goals of SDM. The training emphasized that the goal of SDM is not to get the patient to agree to LCS, but rather to reach the right decision for the individual patient. We role played scenarios in which a patient may reasonably decline LCS because it does not fit with their individual values and goals.

### Original implementation plan: clinical encounter level

The original implementation plan consisted of the implementation strategy *Conduct Outreach Visits* at the clinical encounter level. We planned to conduct these outreach visits, also called academic detailing visits, with LCS coordinators.

### Clinical encounter-level barriers

Participants described several barriers to SDM at the encounter-level: 1) Perception that SDM is already happening; 2) Limiting information about the harms of screening; 3) Lack of decision aids.

#### Perception that SDM is already happening

A clinician expressed confidence that PCPs are engaging in SDM: *“I think all our providers are well-trained and well-versed in talking to patients about the pros and cons about whatever is being done. I don’t think there’s a lack of that.”* – PCLead02.

Another participant shared a script with our team that was described as promoting SDM. Instead, the script prompted the clinician to confirm LCS eligibility and provide information about LCS. Lacking were elements of SDM such as deliberation. The script concluded with the question, “Is it OK to put in the order for the cat scan?” underscoring that the goal of the conversation was to sign patients up for LCS.

#### Limiting information about the harms of screening

Clinicians described the information they provided patients during conversations about LCS. They emphasized the benefits of LCS and were careful not to share information that might make patients hesitant about screening. This included avoiding discussing the possibility of a biopsy if a cancer was found*: “There’s a fine line between explaining all it entails and scaring them, because ultimately we are trying to diagnose cancer at its earliest stage”* – LCSC02.

#### Lack of decision aids

Clinicians noted they did not routinely use decision aids or personalized risk calculators, two tools recommended by multiple organizations, including VA, for facilitating SDM. Some were unfamiliar and others described decisions aids as inconvenient: *“Clinic is not set up in such a way that the decision aids are easily accessible, either paper or web-based. Because I do know it pretty well, I have my schtick down, I often don't use the decision aid.”* – LCSLead02.

Still others saw these tools as unnecessary or not aligned with their workflow*: “I don't know if anyone else is using any [decision aids] around here. I haven’t. I’ve done this for six years. I know how my spiel goes."* – LCSC01.

### Clinical encounter-level tailoring

To address the perception that SDM is straightforward and already happening, the curriculum of our strategy *Conduct Outreach Visits*, also known as academic detailing visits, was tailored. The outreach visits were tailored to address clinician-specific concerns and demonstrate how SDM may change depending on the individual patient. We included opportunities in outreach visits for clinicians to practice SDM one-on-one. To address the minimization of information about the harms of screening and the lack of decision aids, we also introduced a personalized, web-based LCS decision support tool that can be used at the point-of-care. ScreenLC.com [40, 41] was presented to LCS coordinators individually during the outreach visit.

### Original implementation plan: patient level

The original implementation plan consisted of the implementation strategy *Distribute Educational Materials* at the patient level. We planned to adapt and distribute an existing LCS decision aid.

### Patient-level barriers

Clinicians described two perceived barriers to SDM at the patient level: 1) Patients not actively engaging in the LCS conversation; 2) Patients’ smoking history making LCS compulsory.

#### Patients not actively engaging in LCS conversations

Clinicians described patients as generally agreeing to LCS: *“Many [patients] are fairly good at being told what to do by their PCP, say ‘get this screening’ and they show up.”* – LCSC01, demonstrating a lack of SDM with the patient.

Another clinician noted lack of patient engagement in SDM, even after they described LCS: *“Obviously we talk about risks and benefits, but I’ve had very few people decline. They’re just like ‘ok, sure’”* – LCSC08.

#### Patients’ smoking history making LCS compulsory

Participants also raised patients’ smoking history as the rationale to bypass SDM and proceed directly to LCS, despite guidelines recommending SDM for all patients eligible for LCS: *“It’s more like ‘You are a smoker or you were a smoker and you are at high risk for cancer, we would like to do an annual scan to find out if you have it’”* – LCSC02.

### Patient-level tailoring

To address the perception that patients do not want to engage in SDM, we worked directly with patients by incorporating two new ERIC strategies into our implementation plan. We *Involved Patients* and *Developed Educational Materials*, co-designing these materials with patients and clinicians to explicitly empower patients to ask questions and engage in SDM for LCS. We also address smoking stigma explicitly in patient-facing materials.

## Discussion

Our formative evaluation identified barriers to SDM for LCS. We used this information to tailor our WISDOM LCS implementation plan for VA New England. Before commenting on our use of tailoring, however, several of the barriers themselves merit discussion.

We found barriers to SDM for LCS at the organization, clinician, clinical encounter, and patient levels across all facilities. Many of these have been previously identified, especially barriers at the clinician and clinical encounter levels, such as not having time for SDM [22, 42, 43].

Across the VA New England Healthcare System, LCS was not consistently a priority relative to other clinical issues. This made implementing SDM for LCS challenging. This lack of prioritization of LCS could explain why the eighth VA facility, where we did not conduct interviews, lacked an LCS program, despite an offer from regional leadership to fund an LCS coordinator. We noted a tension between SDM and LCS at the organizational level. The current focus on increasing LCS uptake may undermine SDM implementation, though these efforts are not inherently working at cross purposes. Further, uncertainty about the future of the funding for the LCS coordinator role could tip the balance towards wanting to demonstrate strong LCS uptake, which is less complex to measure than SDM. LCS coordinators interviewed described their concern that, if they did not screen enough people for lung cancer, they would not be able to justify the funding for their role. These concerns highlight a larger issue; it is more difficult to routinely measure quality of SDM in comparison to counting the number of LCS orders placed. Services that are more easily measured may be perceived as more valuable.

At the clinician-level, we found a “get the cancer” mentality. There is a strong pro-screening bias in the United States, which clinicians typically share [44]. SDM is predicated on clinicians sharing their medical expertise to inform patients that LCS offers an opportunity to detect lung cancer at an earlier, more treatable stage. However, it is also important that clinicians share the potential harms of screening, such as radiation exposure or overdiagnosis. Thus, it is important that clinicians elicit and consider patients’ values and goals when deciding whether LCS is the right decision for that individual patient. It may be difficult for some clinicians to balance their mental model of their role to keep patients healthy while also respecting patient values and goals, which may sometimes conflict with what the clinician would choose for the patient [45]. Participants alluded to an assumption that SDM would prevent patients from agreeing to LCS, and because clinicians believed so strongly in the benefits of LCS, they did not want to engage in SDM and risk having patients decline screening. It’s difficult to ascertain the impact of SDM on LCS uptake. However, a study by Volk and colleagues found that patients who received a LCS decision aid, which presents both benefits and harms of LCS, were no less likely to be screened for lung cancer than patients who received basic patient education materials [45, 46]. While decision aids strive to present balanced information in a format patients can understand, decision aids are not routinely used to support SDM for LCS in practice, despite multiple studies finding that they increase patient knowledge [47–50].

Clinicians’ pro-screening bias can be seen in the mission to “educate patients” until they agree to LCS. Framing SDM as purely patient education positions the patient as ignorant and the provider as possessing all necessary information, an orientation rooted in paternalism, as the clinician is sharing information, not power [51]. This framing does not allow patients to bring knowledge of their own lives and bodies into the conversation. Because the clinician is the expert in this scenario, following their recommendation is the “right” decision. This may lead to clinicians continuing to educate patients until they make the “right” decision. A paternalistic approach to decision-making disproportionately impacts people of color and other marginalized groups, given the history of racism and oppression of these groups [52, 53]. Work specifically focusing on empowering Veterans of color to engage in SDM for LCS is the focus of a parallel project by our team, funded by VA’s Office of Health Equity [54, 55].

Clinicians also exhibited bias against patients with a smoking history. Clinicians believed that needing to be screened for cancer is the natural consequence of smoking, and precluded patients from making future medical decisions. Clinicians’ stigmatization of people who smoke or smoked is well documented; fortunately, good patient-provider communication can lessen smoking-related stigma [56–58]. Smoking-related stigma, along with pro-screening bias, can inhibit high-quality SDM. Clinicians may not spend time discussing LCS if they do not believe there is truly a decision to be made.

We mapped the barriers identified in our data to levels of the socio-ecological model. We then tailored our multi-level plan to implement SDM for LCS in VA New England. Following the Miller et al. framework, FRAME-IS, for recording and reporting modifications to implementation strategies, we described what is being modified and the nature of the modifications. The goal of the modification was twofold: (1) we made changes to increase the acceptability, appropriateness, and feasibility of the implementation effort and (2) we made changes to improve the sustainability of SDM for LCS, such that the practice continues after our project ends.

A strength of our work is that we proactively tailored our implementation plan by conducting a formative evaluation. To our knowledge, this is the first application of the FRAME-IS to an ongoing implementation project. The modifications made were proactive, rather than reactive, and the rapid, pragmatic approach to tailoring the implementation plan allowed us to improve our current work, rather than waiting for a future implementation project to put the changes into effect. For this to be possible, researchers must reserve resources for formative evaluation work and expect that changes to their implementation plan will be necessary.

Our study has limitations. We did not interview patients as part of this formative evaluation; therefore, we only present clinicians’ perceptions of patients. Understanding patients’ experiences of LCS conversations is the focus of our future work as we implement SDM for LCS. Our findings may be unique to VA or VA New England. One distinction between VA and other healthcare systems is the CMS requirement of SDM for LCS for reimbursement, which only affects non-VA settings but not VA. Although SDM is not required for LCS in the VA, it is notable that studies show that SDM is not occurring routinely outside of VA either [23]. Thus projects like ours that seek to implement tailored strategies to promote SDM for LCS are essential both in and outside VA [17].

Many implementation scientists agree that tailoring implementation strategies is a strength and a necessity to carry out successful implementation work [30, 59]. Tailored implementation plans improve the fit of an implementation effort to a specific context and audience, increasing their success. As such, we anticipate our tailored implementation plan will be more successful than it otherwise would have been without our formative evaluation and subsequent tailoring. Further, planned modification, in comparison to reactive modification, can be a stronger and more thoughtful approach to implementation efforts, highlighting the importance of formative evaluation work in implementation science.

## Conclusions

Our formative evaluation using an ethnographic approach, which informed modifications to the implementation, can be used across implementation science studies. This work demonstrates how FRAME-IS can be used to guide and document modifications to reshape an implementation plan based on contextual barriers identified during a formative evaluation. Our future work will focus on measuring the outcomes of the implementation effort, namely, the frequency and patient-perceived quality of SDM for LCS, to evaluate this implementation plan. Tailoring the implementation plan to local contextual barriers identified through ethnographic data collection methods, guided by application of the FRAME-IS to our formative evaluation, represents an advance both for implementation science methods in general as well as implementation of SDM specifically.

## Data Availability

The datasets generated and/or analyzed during the current study are not publicly available due to them containing information that could compromise research participant privacy but are available from the corresponding author on reasonable request.

## Abbreviations

ATS: American Thoracic Society
CMS: Centers for Medicare and Medicaid Services
ERIC: Expert Recommendations for Implementing Change
FRAME-IS: Framework for Reporting Adaptations and Modifications to Evidence-based Implementation Strategies
IRB: Institutional Review Board
LCS: Lung Cancer Screening
LDCT: Low Dose Computed Tomography
PCP: Primary Care Provider
SDM: Shared Decision-Making
USPSTF: United States Preventive Services Task Force
VA: Department of Veterans Affairs
WH: Whole Health
WISDOM LCS: Whole Health Approach to Implementing Shared Decision Making for Lung Cancer Screening
